# The transition to parenthood following a history of childhood maltreatment: a review of the literature on prospective and new parents’ experiences

**DOI:** 10.1080/20008198.2018.1492834

**Published:** 2018-08-02

**Authors:** Hope Christie, Anat Talmon, Sarah Katharina Schäfer, Anke de Haan, Maria Louison Vang, Katharina Haag, Ohad Gilbar, Eva Alisic, Erin Brown

**Affiliations:** a Department of Psychology, University of Bath, Bath, UK; b The Bob Shapell School of Social Work, Tel Aviv University, Tel Aviv, Israel; c Department of Clinical Psychology and Psychotherapy, Saarland University, Saarbrücken, Germany; d Department of Psychology – Child and Adolescent Health Psychology, University of Zurich, Zurich, Switzerland; e School of Psychology, Ulster University, Coleraine, UK; f School of Social Work, Bar-Ilan University, Ramat Gan, Israel; g Accident Research Centre, Monash University, Melbourne, Australia; h Melbourne School of Population and Global Health, University of Melbourne, Melbourne, Australia; i School of Psychology, University of Queensland, St Lucia, Australia

**Keywords:** Adverse childhood experiences, childhood abuse, fatherhood, motherhood, parenting, pregnancy, Experiencias infantiles adversas, maltrato infantil, paternidad, maternidad, crianza, embarazo, 不良儿童经历, 童年虐待, 父亲, 母亲, 为人父母, 怀孕, • This study reviewed 69 papers to further understand the experiences of prospective and new parents’ transition to parenthood in the context of a history of childhood maltreatment.• Having a history of maltreatment may be a risk factor for a more challenging transition to parenthood.• Parents with maltreatment histories may suffer from a range of mental health difficulties during their transition to parenthood.• Women with maltreatment histories may also suffer from negative physical changes during pregnancy and postpartum.• During the transition to parenthood, parents with maltreatment histories may also experience negative views of the child and of themselves as parents.

## Abstract

**Background:** Becoming a parent is viewed as one of the most important transitions in one’s life. However, a history of childhood maltreatment may affect the adjustment to parenthood.

**Objective:** The objective of this review was to synthesize the current evidence base to further our understanding of prospective and new parents’ experiences in the transition to parenthood (pregnancy to 2 years post-birth), in the context of having a childhood maltreatment history.

**Method:** A scoping review of the literature was conducted using the following online databases: PubMed, PsycINFO, PsycNET, and Published International Literature of Traumatic Stress.

**Results:** The findings were synthesized into a four-component theoretical framework, which included mental health of the parent, physical changes, parental view of the child, and view of the self as a parent. A total of 69 papers, including 181,537 participants (of whom 30,482 mothers and 235 fathers had maltreatment histories), investigated the transition to parenthood. The majority of the studies showed that parents with a maltreatment history may suffer from a range of mental health problems during the transition to parenthood, experience more negative physical changes, and have more negative views of their child (or children). However, they reported both positive and negative experiences regarding their identity as a parent.

**Conclusions:** The findings suggest that maltreatment is a risk factor for a more challenging transition to parenthood. Experiences of fathers with maltreatment histories merit more attention, as do those of parents in low- and middle-income countries. Future directions should include predictors of positive experiences and the development of early interventions to improve outcomes for this population.

## Abstract (es)

**Antecedentes**: Convertirse en padre es visto como una de las transiciones más importantes en la vida. Sin embargo, un historial de abuso infantil puede afectar la adaptación a la paternidad.

**Objetivo**: El objetivo de esta revisión fue sintetizar la evidencia actual para ampliar nuestra comprensión de las experiencias de los nuevos y futuros padres en la transición a la paternidad (desde el embarazo a dos años después del nacimiento), en el contexto de tener un historial de abuso infantil.

**Método**: se realizó una revisión de la literatura utilizando las siguientes bases de datos en línea: PubMed, PsycINFO, PsycNET y Literatura Internacional Publicada de Estrés Traumático.

**Resultados**: los hallazgos se sintetizaron en un encuadre teórico de cuatro componentes, que incluyó la salud mental de los padres, los cambios físicos, la visión del niño por parte de los padres y la visión de uno mismo como padre. Un total de 69 artículos, que incluían 181,537 participantes (30,482 madres y 235 padres con antecedentes de abuso), investigaron la transición a la paternidad. La mayoría de los estudios mostraron que los padres con antecedentes de abuso pueden experimentar una variedad de problemas de salud mental durante la transición a la paternidad, experimentan más cambios físicos negativos y tener una opinión más negativa de su(s) hijo(s). Sin embargo, informaron experiencias tanto positivas y negativas con respecto a su identidad como padre.

**Conclusiones**: los hallazgos sugieren que el abuso es un factor de riesgo para una transición más compleja a la paternidad. Las experiencias de los padres con historias de abuso merecen mayor atención, al igual que los padres en países de bajos y medianos ingresos. Las direcciones futuras debieran incluir predictores de experiencias positivas y desarrollo de intervenciones tempranas para mejorar los resultados para esta población.

## Abstract (zh)

背景：成为家长被认为是人生中最重要的转折点之一。然而，童年虐待的历史可能会影响对为人父母角色的适应。

目的：本综述的目的是综合当前的证据基础，以进一步理解有童年虐待历史的准父母和新父母在向父母身份过渡（从怀孕到出生后两年）过程中的经历。

方法：使用以下在线数据库对文献进行范围审查：PubMed，PsycINFO，PsycNET和已发表的创伤应激国际文献。

结果：研究结果被综合为有四个部分的理论框架，包括父母的心理健康状况、身体变化、父母对孩子的看法。以及对自身作为父母的看法。共有检索到69篇论文，其中包括181,537名参与者（有虐待历史的30,482名母亲和235名父亲），调查了其向为人父母过渡的情况。大多数研究表明，有虐待史的父母可能在向为人父母过渡期间遭受一系列心理健康问题，经历更负面的身体变化，并对其子女有更多的负面看法。然而，他们同时报告了对父母身份的正面和负面体验。

结论：研究结果表明，虐待是使得向父母过渡更具挑战性的一个风险因素。有虐待历史的父亲的经历更值得关注，在低收入和中等收入国家的父母也是如此。未来的方向应包括积极经验的预测指标和早期干预措施的发展，以帮助此人群进行改善。

## 1. Introduction

The transition to parenthood can involve aspects of growth and development, as well as anxiety about becoming a parent. Managing the impact of change following the birth of the baby and subsequent adjustment to new roles within the family unit can also be challenging (Cowan & Cowan, 1995; Deave, Johnson, & Ingram, 2008). These demands of the transition to parenthood have been conceptualized and investigated largely in terms of mental health outcomes (Schulz, Cowan, & Cowan, 2006), and difficulties with parenting behaviour independent from a history of childhood maltreatment (Berg-Nielsen, Vikan, & Dahl, 2002). However, parents who have a history of childhood maltreatment (subsequently referred to as ‘maltreatment history’) may experience unique challenges when transitioning to parenthood (DiLillo & Damashek, 2003).

Childhood maltreatment is an umbrella term for childhood sexual abuse, physical abuse, emotional abuse, physical neglect, and emotional neglect (Stoltenborgh, Bakermans-Kranenburg, Alink, & van Ijzendoorn, 2015). A maltreatment history is commonly reported by both clinical and non-clinical populations (Perez-Fuentes et al., 2013). A recent meta-analysis reported high percentages of childhood maltreatment in non-clinical populations (23% physical abuse, 13% sexual abuse, 36% emotional abuse, 16% physical neglect, and 18% emotional neglect; Stoltenborgh et al., 2015). The high prevalence indicates that a large proportion of adults might find the transition to parenthood challenging.

Several reviews have shown that childhood maltreatment is negatively related to socioeconomic status (Currie & Widom, 2010), as well as physical and mental well-being (Messman-Moore & Bhuptani, 2017; Norman et al., 2012). As childhood maltreatment is prevalent and becoming a parent is a central component in many people’s lives, it is important to investigate whether and how a maltreatment history influences adjustment to parenthood. Research on parents with maltreatment histories has primarily focused on: adverse obstetric outcomes (Blackmore et al., 2016); implications for infant development (Buss et al., 2017); the detrimental impact of abuse on parenting abilities (DiLillo & Damashek, 2003); and the intergenerational ‘cycle of abuse’, the mechanism which ‘turns’ a maltreated child into an abusive parent (Dixon, Browne, & Hamilton-Giachritsis, 2005). Adults with a maltreatment history may face a unique experience in transitioning to parenthood (Roberts, 2014), and a greater understanding of these experiences may aid professionals in supporting this vulnerable population.

Theories of the transition to parenthood have been proposed by Raphael-Leff (2001) and Stern and Bruschweiler-Stern (1998). These frameworks detail three sequential phases that correspond to the trimesters of pregnancy. Phase one focuses on the physical and biological changes in the prospective mother (Sternberg & Blinn, 1993). Phase two highlights the relationship to the foetus/baby during mother–foetus bonding. This period is characterized by the mother-to-be’s hopes and dreams regarding her baby (Siddiqui & Hagglof, 2000). Phase three details the creation of a new identity as a mother, as well as expectations, anxieties, and goals regarding this role. This period may involve the mother-to-be being exposed to new aspects of herself that require reconstructing her identity (Bailey, 1999).

Raphael-Leff (2001) and Stern and Bruschweiler-Stern (1998) focused on the transition to motherhood and its psychological aspects, while Genesoni and Tallandini (2009) categorized the transition to fatherhood into three main elements: first, the image of the self as a father; secondly, the bonding with the baby and the dynamic in the mother–father–child triad; and lastly, vulnerability to potential outer voices from the environment and surroundings. While there is evident overlap between these frameworks for mothers and fathers, they are yet to be considered in relation to parents with maltreatment histories.

For the purposes of the current review, Raphael-Leff’s (2001) and Stern and Bruschweiler-Stern’s (1998) frameworks were utilized to provide an a-priori theoretical lens to guide the screening and synthesizing of the literature investigating the transition to parenthood (rather than motherhood and fatherhood) for those with maltreatment histories. The timeline of the phases (i.e. associated with trimesters) was removed, as the different phases described seemed applicable across the entire pregnancy and postnatal period, rather than only in the trimesters. Therefore, the current evidence base was synthesized in terms of (1) physical changes; (2) the view of the foetus/child; and (3) the view of self as a parent.

The aim of this scoping review was to identify current available knowledge on prospective and new parents’ experiences in the transition to parenthood following a maltreatment history. The above model provided a theoretical lens through which to understand and synthesize the evidence.

## 2. Method

### 2.1. Literature search strategy

**Figure 1.**
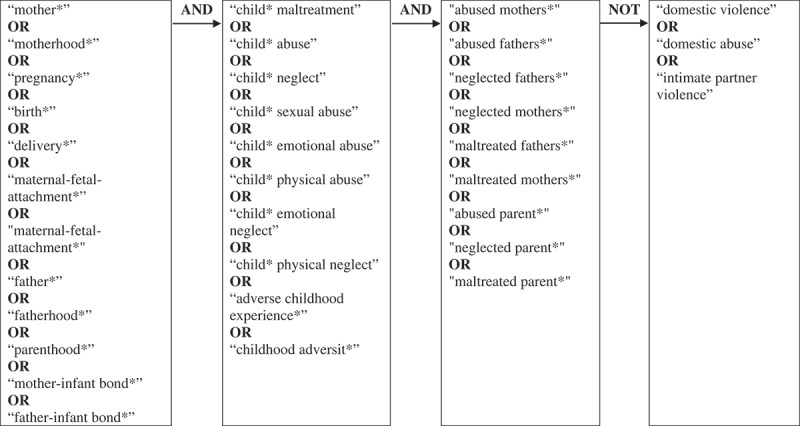
Summary of the search terms used in the web-based search.

**Figure 2.**
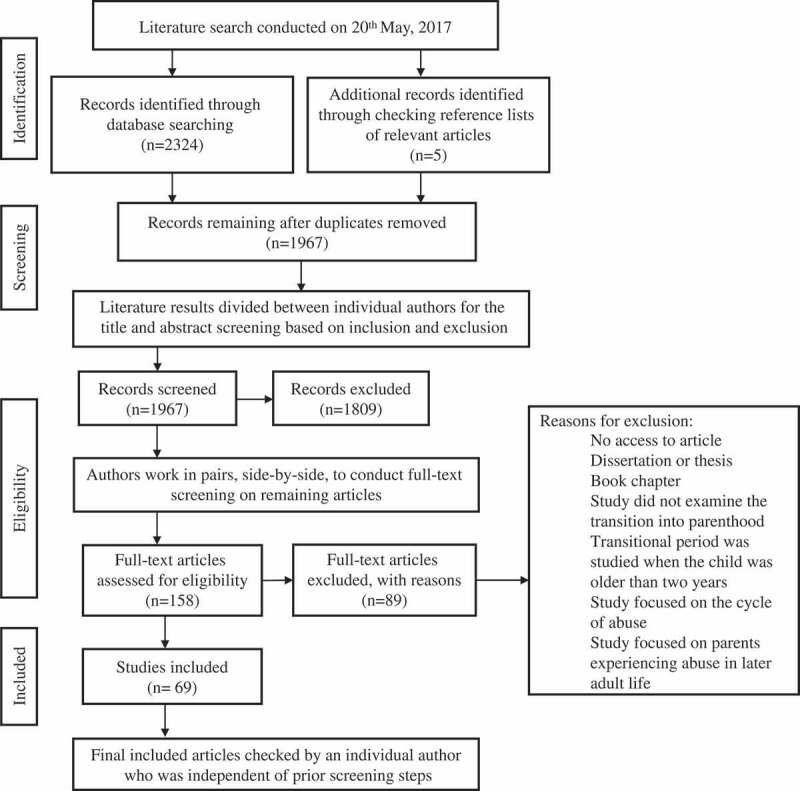
Flowchart of the study screening and selection process.

Searches were conducted using PubMed, PsycINFO, PsycNET, and Published International Literature of Traumatic Stress. Search terms can be found in Figure 1. The literature search was explorative and not constrained or led by the theoretical framework. After consulting a university librarian, search term engine applications such as ‘term finder’ (PsycNET) or ‘MeSH terms’ (PubMed) were not used, in order to ensure consistency across all databases. Additional papers were found through checking the reference lists of relevant articles. Figure 2 maps the literature review process in a Preferred Reporting Items for Systematic Reviews and Meta-Analyses (PRISMA) flowchart (Moher, Liberati, Tetzlaff, Altman, & group, 2009).

### 2.2. Design of the project

Given the broad and diverse body of literature, a scoping review was chosen to allow the authors to provide a descriptive overview, synthesizing findings from a range of study designs and methodologies (Pham et al., 2014). Since the literature search yielded a large number of papers, the aforementioned modified framework was used to order and synthesize the available literature according to themes previously identified as central to the transition to parenthood (Raphael-Leff, 2001; Stern & Bruschweiler-Stern, 1998).

### 2.3. Eligibility criteria

Following the search, duplicates were removed using EndNote (Thomson Reuters, USA), followed by a manual check. Remaining articles were assessed using the following eligibility criteria. Studies were included if: (1) they were published in English in a peer-reviewed journal; (2) they reported empirical data; (3) their focus was the parents’ transition to parenthood; (4) they included a parent or parents who had a maltreatment history; and (5) the participants’ children were under the mean age of 2 years old, as this period is identified as being the most crucial transitioning period for parenthood and for healthy child development (Public Health England, 2016). Included studies examined parents with a maltreatment history only, or maltreated parents with a control group of non-maltreated parents, in which case the relative effect of childhood maltreatment was reported. The following contributions were excluded: (1) book chapters, dissertations, or reviews; (2) papers focusing solely on the cycle of abuse, as the focus was on parents’ experiences, rather than predicting intergenerational abuse; and (3) samples with adult maltreatment but not a maltreatment history.

### 2.4. Data extraction

Following the literature search, the authors screened articles and extracted data. To ensure that no articles were wrongfully excluded and all information extracted was accurate, the authors worked in pairs to monitor consistency and reliability. Finally, all included papers and extracted information were reviewed by an additional author not involved in the first process. The step-by-step process is shown in the literature search flowchart in Figure 2. Prior to the screening, all authors screened 15 papers to assess reliability and consistency. The results indicated a 100% level of agreement regarding eligibility of studies. To ensure the accuracy of the information extracted from the included studies, data were initially extracted by one of the authors, before being reviewed by a secondary coder. Discrepancies were discussed between the authors and agreement was reached in all cases.

## 3. Results

Throughout screening, it was evident that much of the research examined the mental health of prospective and new parents with a maltreatment history. The authors identified this as an important aspect of the transition, despite its absence from the theoretical framework. Therefore, the framework was modified to include mental health manifestations as a fourth component that was central to the other three components.

A total of 69 papers (52 different samples) investigated the transition to parenthood following childhood maltreatment (Table 1). The majority of papers involved samples from North America (80%), followed by Europe (16%), Asia (3%), and Africa (1%). The total number of participants across papers was calculated excluding repeated samples (when repeated sample sizes differed, the largest sample size was included). The current analysis builds on data from 181,537 participants, of whom approximately 30,717 had maltreatment histories (30,482 mothers and 235 fathers). Since 2009, this area has received increased research interest, with the highest number of papers published between 2015 and 2016 (32%). The majority of studies recruited parents with a history of unspecified childhood maltreatment (83%); five studies recruited parents with a history of physical maltreatment only and four studies sexual maltreatment only.

**Table 1.** Characteristics of the included research papers.

Authors (year)	Country	Childhood maltreatment cases/total *N*^a^	Parental age, M (range)	Parenting stage (years;months)	Recruitment/sample characteristics	Type(s) of maltreatment
Altemeier et al. (1986)	USA	95/927	20	Child 1;21–48 months old	Low-income mothers-to-be, recruited from prenatal clinic or inner-city hospital	Childhood maltreatment (physical)
Aparicio et al. (2015)	USA	6/6	21 (19–22)	Gestation (retrospective) Child 1–7 years old (current)	Teen mothers in foster care	Childhood maltreatment (any)
Berlin et al. (2011)	USA	48/499	27	18–36 weeks’ gestation (52% primiparous^b^)	Community-based study on mothers-to-be	Childhood maltreatment (physical)
Bernazzani and Bifulco (2003)	UK	198	34 (20–45)	Gestation (retrospective)	Community-based study on mothers	Childhood maltreatment (any)
Bernstein et al. (2013)	USA	105	24 (18–37)	T1: 28–40 weeks’ gestation T2: Child 5 months old T3: Child 1;7 months old	Mothers-to-be recruited from childbirth education classes, hospitals, and public assistance organizations	Childhood maltreatment (any)
Berthelot et al. (2015)	Canada	57/57	29 (19–41)	T1: 28–40 weeks’ gestation T2: Child 17 months old	Mothers-to-be recruited through obstetrics clinic at hospital	Childhood maltreatment (any)
Bouvette-Turcot et al. (2017)^c^	Canada	230	Not given	T1: 24–36 weeks’ gestation T2: Child 6 months old	Community-based study on mothers-to-be	Maternal adversity, including childhood maltreatment (any)
Bouvette-Turcot et al. (2015)^c^	Canada	154	33	T1: Child 1;6 years old T2: Child 3 years old T3: Child 5 years old	Community-based study on mothers	Maternal adversity, including childhood maltreatment (any)
Brand et al. (2010)	USA	38/126	34	Child 6 months old	Community-based study on mothers	Childhood maltreatment (any)
Bublitz and Stroud (2013)^d^	USA	27/41	26	T1: 13–27 weeks’ gestation T2: 27–40 weeks’ gestation	Community-based study on mothers-to-be	Childhood maltreatment (any)
Bublitz et al. (2014)^d^	USA	185	26 (18–40)	T1: 25 weeks’ gestation T2: 29 week’s gestation T3: 35 weeks’ gestation	Community-based study on mothers-to-be	ACEs
Caldwell et al. (2011)	USA	76	28 (18–44)	Child 0;1–16 years old	Community programme for mothers deemed at risk	Childhood maltreatment (any)
Casanueva et al. (2010)	USA	249/1001	(14+)	Child 0–2 years old	Data from national health survey, and mothers under investigation for child maltreatment	Childhood maltreatment (any)
Cederbaum et al. (2013)	USA	20,732/153,762	(12–19)	Child 0 years old (birth)	Mothers analysed from child’s birth records	Childhood maltreatment (any)
Choi and Seng (2016)^e^	USA	110/564	27	T1: Initiation of prenatal care T2: 35 weeks’ gestation T3: Child 1 month old	Primiparous mothers-to-be recruited through prenatal clinics	Childhood maltreatment (any)
Choi et al. (2015)	South Africa	35/84	Not given	26 weeks’ gestation	Mothers-to-be attending an antenatal clinic in a high HIV risk area	Childhood maltreatment (any)
Christiaens et al. (2015)	Canada	75/223	28	Child 0;3–1 year old	Mothers recruited from hospitals	ACEs
Chung et al. (2009)^f^	USA	961/1265	24 (14–44)	T1: Child 3 months old T2: Child 11 months old	Mothers-to-be attending prenatal care services	Childhood maltreatment (any) and/or indirect exposure to violence
Chung et al. (2010)^f^	USA	1055/1476	24	T1: 14 weeks’ gestation T2: Child 3 months old T3: Child 11 months old	Mothers-to-be attending prenatal care services	Childhood maltreatment (any), indirect exposure to violence, and/or unstable/unsafe living environment
Dayan et al. (2010)	France	693	29 (18–45)	20–28 weeks’ gestation	Mothers-to-be recruited from hospital	Childhood maltreatment (any)
Dayton et al. (2016)^g^	USA	78/120	26 (18–42)	T1: 24–36 weeks’ gestation T2: Child 1 year old T3: Child 2 years old	Advertisements, agencies serving low-income mothers-to-be	Childhood maltreatment (any)
Dietz et al. (1999)	USA	785/1193	(20–50)	Gestation (retrospective)	Mothers who attended a clinic during 1995–1996	Childhood maltreatment (any), indirect exposure to violence, and/or unstable/unsafe living environment
Drevin et al. (2015)	Sweden	88/142	31 (30–31)	T1: 6–13 weeks’ gestation T2: 31–36 weeks’ gestation	Mothers recruited from antenatal clinics	ACEs
Dym Bartlett and Easterbrooks (2015)	USA	206/447	19	Child 0;2–2;5 years old	Visiting programme for primiparous young mothers	Childhood maltreatment (any)
Erdmans and Black (2008)	USA	27/27	20	Child 0–5 years old	Visiting programme for primiparous mothers at risk of abusing child	Childhood maltreatment (sexual)
Esparza et al. (1996)	USA	54/124	(13–20)	Gestation (56% primiparous)	Agencies caring for adolescent African-American and Mexican-American mothers	Childhood maltreatment (sexual)
Farber et al. (1996)	USA	103/309	27 (14–40)	8–40 weeks’ gestation	Low-income mothers-to-be attending psychiatry–obstetrics service	Childhood maltreatment (any)
Fava et al. (2016)	USA	100/100	30	Child 6 months old	Mothers recruited from advertisements in obstetric clinics	Childhood maltreatment (any)
Fogel and Belyea (2001)	USA	63	(16–39)	32–40 weeks’ gestation	Mothers-to-be in prison	Childhood maltreatment (any)
Frankenberger et al. (2015)	USA	1386/1987	(18+)	Gestation (retrospective)	Data from annual health survey	ACEs
Fuchs et al. (2016)	Germany	41/88	32	Child 1 year old	Mothers attending obstetric clinic	Childhood maltreatment (any)
Gara et al. (2000)	USA	38/74	21	T1: Child 6 months old T2: Child 1 year old T3: Child 2 years old	Mothers recruited from community health clinics, in two early prevention/baby nutrition programmes	Childhood maltreatment (physical)
Gonzalez et al. (2012)	Canada	27/89	32 (24–42)	Child 0.2–0.5 years old	Community sample of mothers recruited from health centres and hospitals	Childhood maltreatment (any)
Herzog et al. (1992)	USA	3/5	(18–20)	T1: Child 3 months old T2: Child 1 year old T3: Child 2 years old	Mothers part of home-visiting programme	Childhood maltreatment (any)
Huth-Bocks et al. (2013)^g^	USA	78/120	(18–42)	24–36 weeks’ gestation	Advertisements, agencies serving low-income mothers-to-be	Childhood maltreatment (any)
Kettunen and Hintikka (2017)	Finland	67/208	30 (18–40)	Child 1–6 months old	Mothers attending antenatal clinics	Childhood maltreatment (any) and/or indirect exposure to violence
Lang et al. (2010)^h^	USA	44	29	T1: 7–32 weeks’ gestation T2: Gestation (unspecified) T3: Gestation (unspecified) T4: Child 0 years old T5: Child 1 year old	Mothers-to-be recruited from advertisements in obstetric clinics	Childhood maltreatment (any)
Lang et al. (2006)^h^	USA	44	29	T1: 7–32 weeks’ gestation T2: Child 1 year old	Mothers-to-be recruited from advertisements in obstetric clinics	Childhood maltreatment (any)
Leeners et al. (2014)	Germany	85/255	27	Gestation (retrospective)	Mothers enrolled in programme for sexual abuse support, and recruited from kindergartens/GP surgeries	Childhood maltreatment (sexual)
Lev-Wiesel et al. (2009)	Israel	323/837	31 (18–44)	T1: 17–30 weeks’ gestation T2: Child 2 months old T3: Child 6 months old	Convenience sample of Jewish mothers	Childhood maltreatment (any)
Li et al. (2017)	China	129/275	26	T1: 28+ weeks’ gestation T2: Child 0 years old (birth) T3: Child 1 month old	Mothers-to-be recruited from hospital	Childhood maltreatment (any)
Lyons-Ruth et al. (1996)^i^	USA	45	Not given	Child 1;6 years old	Low-income mothers with or without referral to social services	Childhood maltreatment (any)
Lyons-Ruth et al. (1989)^i^	USA	50	26	T1: Child 1 year old T2: Child 1;6 years old	Low-income mothers with or without referral to social services	Childhood adversity, including childhood maltreatment (physical)
Malone et al. (2010)	USA	204	25 (18–40)	28–40 weeks’ gestation	Mothers-to-be recruited through health clinics and community advertisements	Childhood maltreatment (any)
Marcenko et al. (2000)	USA	68/127	27 (13–42)	Child < 3 years old	Low-income, African-American mothers with current or history of substance abuse, incarceration, children in out-of-home placement, HIV-positive, domestic violence, and/or late/no prenatal care	Childhood maltreatment (any)
Martinez-Torteya et al. (2014)^j^	USA	103/153	29 (18–45)	Child 0.6 years old	Mothers attending prenatal care clinics, or recruited through community advertisements, oversampled for childhood trauma	Childhood maltreatment (any)
Marysko et al. (2010)	Germany	58/119	Not given	T1: Child 5 months old T2: Child 1 year old	All mothers-to-be in local area were contacted by mail	Childhood maltreatment (any)
McDonnell and Valentino (2016)	USA	192/398	25 (15–46)	T1: M = 16 weeks’ gestation T2: Child 6 months old	Community sample of mothers-to-be	Childhood maltreatment (any)
Milan et al. (2004)	USA	146/203	17	Child 1 year old	Mothers with HIV/STI risk behaviours, attending hospital clinics	Childhood maltreatment (physical)
Michl et al. (2015)	USA	83/127	25 (18–38)	Child 1 year old	Non-clinical, low-income mothers attending primary care clinics	Childhood maltreatment (any)
Morelen et al. (2016)^j^	USA	137/192	29 (18–45)	Child 6 months old	Mothers attending prenatal care clinics, or recruited through community advertisements, oversampled for childhood trauma	Childhood maltreatment (any)
Muzik et al. (2013)^j^	USA	97/150	29	T1: Child 1 months old T2: Child 4 months old T3: Child 6 months old	Mothers attending prenatal care clinics, or recruited through community advertisements, oversampled for childhood trauma	Childhood maltreatment (any)
Muzik et al. (2017)^j^	USA	102/164	29 (18–45)	Child 6 months old	Mothers attending prenatal care clinics, or recruited through community advertisements, oversampled for childhood trauma	Childhood maltreatment (any)
Muzik et al. (2016)^j^	USA	116/116	29	T1: Child 4 months old T2: Child 6 months old T3: Child 1 year old T4: Child 1;3 years old T5: Child 1;6 years old	Mothers attending prenatal care clinics, or recruited through community advertisements, oversampled for childhood trauma	Childhood maltreatment (any)
Oh et al. (2016)^j^	USA	117	29 (18–45)	T1: Child 0.3 years old T2: Child 6 months old T3: Child 1 year old T4: Child 1;3 years old T5: Child 1;6 years old	Mothers attending prenatal care clinics, or recruited through community advertisements, oversampled for childhood trauma	Childhood maltreatment (any)
Plant et al. (2013)	UK	125/125	26 (17–44)	T1: 36 weeks’ gestation T2: Child 3 months old T3: Child 1 year old T4: Child 4 years old T5: Child 11 years old T6: Child 16 years old	Community study on mothers-to-be	Childhood maltreatment (any)
Roberts et al. (2004)	UK	127/8292	Not given	T1: Child 1;9 years old T2: Child 2;9 years old	Mothers-to-be who gave birth during 1991–1992, recruited through midwives, public advertisements, and direct contact	Childhood maltreatment (sexual)
Schechter et al. (2004)	USA	41/41	30 (19–45)	Child 0;8–4;2 years old	Mothers attending health service, during 2000–2001	Interpersonal violent trauma exposure, including childhood maltreatment (physical or sexual)
Seng et al. (2014)^e^	USA	543/1581	(18–47)	< 28 weeks’ gestation	Primiparous mothers-to-be recruited through prenatal clinics	Childhood maltreatment (any)
Seng et al. (2013)^e^	USA	376/566	27 (18+)	T1: < 28 weeks’ gestation T2: 35 weeks’ gestation T3: Child 1 month old	Primiparous mothers-to-be recruited through prenatal clinics	Childhood maltreatment (any)
Sexton et al. (2017)^j^	USA	126/173	29	Child 6 months old	Mothers attending prenatal care clinics, or recruited through community advertisements, oversampled for childhood trauma	Childhood maltreatment (any)
Sexton et al. (2015)^j^	USA	145/214	28	Child 4 months old	Mothers attending prenatal care clinics, or recruited through community advertisements, oversampled for childhood trauma	Childhood maltreatment (any)
Shea et al. (2007)	Canada	66	30	25–33 weeks’ gestation	Mothers-to-be 12–24 weeks’ gestation presenting to health clinic with symptoms of depression	Childhood maltreatment (any)
Shenk et al. (2017)^k^	USA	220	21 (16–42)	T1: Child 0 years old (birth) T2: Child 6 months old T3: Child 1;3 years old	Primiparous mothers enrolled in a home visiting programme for mothers deemed at risk	Childhood maltreatment (any)
Skjothaug et al. (2015)	Norway	235/881	32 (16–56)	T1: 8–34 weeks’ gestation T2: 20–25 weeks’ gestation T3: 26–31 weeks’ gestation T4: 32–34 weeks’ gestation T5: 36 weeks’ gestation	Fathers-to-be recruited at the health clinic appointments for the mothers-to-be	ACEs
Stacks et al. (2014)^j^	USA	58/83	30	Child 0.6–1.3 years old	Mothers attending prenatal care clinics, or recruited through community advertisements, oversampled for childhood trauma	Childhood maltreatment (any)
Teeters et al. (2016)^k^	USA	220	21 (16–42)	T1: < 28 weeks’ gestation T2: Child 6 months old T3: Child 1;3 years old	Primiparous mothers enrolled in a home visiting programme for mothers deemed at risk	Childhood maltreatment (any)
Ukah et al. (2016)	Canada	363/697	(18–49)	Child 6 months old (retrospective)	Data from national health surveys	ACEs
Van der Waerden et al. (2015)	France	527/1807	30	T1: 24–28 weeks’ gestation T2: Child 4 months old T3: Child 8 months old T4: Child 1 year old T5: Child 3 years old T6: Child 5 years old	Mothers analysed from child’s birth records during 2003–2011	Childhood adversity, including maltreatment (any)

M, mean; ACE, adverse childhood experience; HIV, human immunodeficiency virus; GP, general practitioner; STI, sexually transmitted infection; T1, Time 1; T2, Time 2; T3, Time 3; T4, Time 4; T5, Time 5.

^a ^Reported when given; otherwise, only total sample size is reported.

^b ^Having given birth for the first time.

^c ^MAVAN study: Bouvette-Turcot et al. (2015), Bouvette-Turcot et al. (2017).

^d ^BAMBI study: Bublitz and Stroud (2013), Bublitz et al. (2014).

^e ^STACY study: Seng et al. (2013, 2014), Choi and Seng (2016).

^f ^Same project: Chung et al. (2009, 2010).

^g ^Same project: Dayton et al. (2016), Huth-Bocks et al. (2013).

^h ^Same project: Lang et al. (2006, 2010).

^i ^Same project: Lyons-Ruth et al. (1989, 1996)

^j ^MACY study: Stacks et al. (2014), Morelen et al. (2016), Martinez-Torteya et al. (2014), Muzik et al. (2013, 2016, 2017), Oh et al. (2016), Sexton et al. (2015, 2017).

^k ^Same project: Shenk et al. (2017), Teeters et al. (2016).

**Figure 3.**
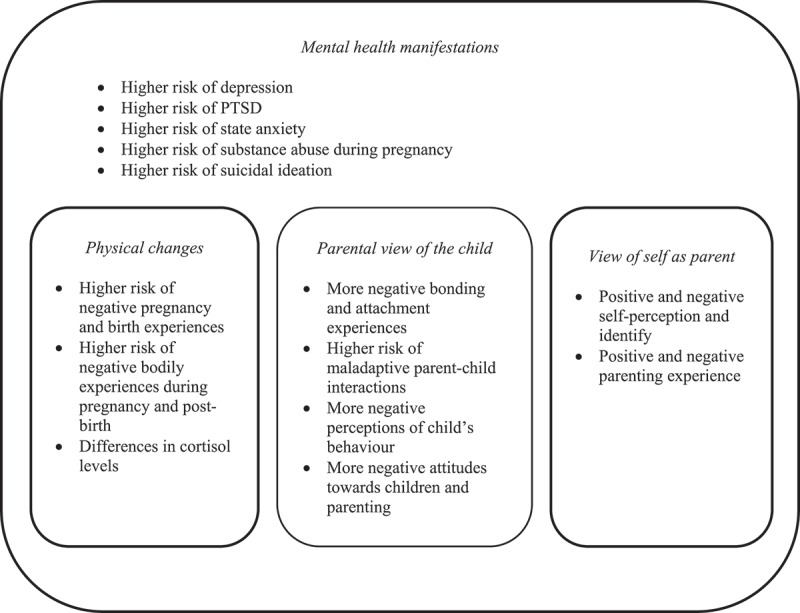
Outline of findings in relation to the model of transition to parenthood (pregnancy and postnatal periods) following childhood maltreatment. PTSD, post-traumatic stress disorder.

Findings pertaining to each phase of the four-component model are outlined in Figure 3 and reported in detail below.

### 3.1. Mental health of the parent

The mental health of prospective and new parents with maltreatment histories received substantial attention in the studies included in the current review (36 articles: 35 among mothers and one among fathers). The most common topics of research were perinatal and postpartum depression (28 studies) and post-traumatic stress disorder (PTSD, 16 studies). Fewer studies investigated anxiety (five studies), substance abuse (four studies), and suicidal ideation (two studies).

#### 3.1.1. Depression

During the perinatal period, women with maltreatment histories were at greater risk for developing depression than those without (McDonnell & Valentino, 2016; Muzik et al., 2013). These results were consistently found across several different samples, including community populations (Bouvette-Turcot et al., 2015), mothers at risk for human immunodeficiency virus (HIV) infection (Choi et al., 2015), and female prisoners (Fogel & Belyea, 2001). In addition, findings were consistent across different types of childhood maltreatment, including sexual or emotional abuse, and physical or emotional neglect (Choi et al., 2015; Leeners, Rath, Block, Gorres, & Tschudin, 2014; Li, Long, Cao, & Cao, 2017). Studies also highlighted factors that may place women at high risk of developing depression during pregnancy, including the use of maladaptive coping strategies, such as avoidance and self-blame (Choi et al., 2015), and maltreatment severity (i.e. increasing number of maltreatment types; Li et al., 2017). Furthermore, maltreatment severity predicted the severity of depression symptoms up to 18 months postpartum (Sexton, Hamilton, McGinnis, Rosenblum, & Muzik, 2015; Teeters et al., 2016), as well as accounting for a smaller reduction in depression symptoms in mothers with prenatal depression symptoms from prepartum to postpartum (McDonnell & Valentino, 2016).

Postpartum, maltreatment histories were associated with new onset of depressive episodes (Kettunen & Hintikka, 2017; Li et al., 2017), and continued to be significantly associated with maternal depression up to 16 months postpartum (Bouvette-Turcot et al., 2015; Lang, Gartstein, Rodgers, & Lebeck, 2010; Martinez-Torteya et al., 2014; McDonnell & Valentino, 2016; Stacks et al., 2014). However, no significant differences were found between postpartum depression and specific maltreatment types (emotional abuse, sexual abuse, and emotional neglect; Li et al., 2017).

Postpartum factors that placed women with maltreatment histories at higher risk for developing depression included: lower levels of resilience (Oh et al., 2016; Sexton et al., 2015), low social support and external locus of control (9–18 months postpartum; Teeters et al., 2016), negative life events during pregnancy, and previous mental health problems (24–28 weeks’ gestation to 5 years postpartum; Van der Waerden, Saurel-Cubizolles, Sutter-Dallay, & Melchior, 2015). Furthermore, Caldwell, Shaver, Li, and Minzenberg (2011) reported that maternal attachment anxiety significantly mediated the relationship between maltreatment history and postpartum depression. One study reported that pre-existing depression alone did not mediate the association between maltreatment history and postpartum depression, whereas PTSD with comorbid depression did mediate this relationship (Seng et al., 2013).

#### 3.1.2. Post-traumatic stress disorder

A maltreatment history consistently predicted post-traumatic stress symptoms (PTSS) during the transition to parenthood (Lang et al., 2010; Martinez-Torteya et al., 2014; Seng et al., 2013). Parents with a maltreatment history exhibited significantly more PTSS during pregnancy and postpartum than parents without a maltreatment history (Muzik et al., 2013; Stacks et al., 2014). Specifically, a sexual and emotional abuse history significantly predicted PTSS during pregnancy (Choi et al., 2015) and postpartum compared to women who had experienced different types of adversities (Lev-Wiesel, Daphna-Tekoah, & Hallak, 2009). Comparatively, parents with physical or emotional abuse histories were more likely to develop PTSD comorbidly with difficulties in affect regulation (Seng et al., 2013).

Maladaptive coping strategies were found to mediate the relationship between maltreatment history and PTSS, after controlling for parental age, gestational age, and HIV status (Choi et al., 2015). During labour, a maltreatment history significantly predicted dissociative experiences, which subsequently explained 20% of the variance in postpartum PTSD (Choi & Seng, 2016). High levels of comorbid PTSD and depression were reported in samples with a history of childhood sexual abuse (Seng et al., 2013) and with more severe maltreatment histories (Oh et al., 2016).

#### 3.1.3. Anxiety

A sexual abuse history predicted significantly higher levels of anxiety symptoms, compared to mothers without a sexual abuse history (Roberts, O’Connor, Dunn, & Golding, 2004). Other research investigated the relationship between maternal maltreatment history and state/trait anxiety (Lang et al., 2010). Significant positive correlations were found between state anxiety and maltreatment history (emotional/sexual abuse, emotional/physical neglect), but not trait anxiety.

#### 3.1.4. Substance abuse

Severity of maltreatment history was positively associated with an increased risk for alcohol use during pregnancy in a representative sample of US women (retrospective report; Frankenberger, Clements-Nolle, & Yang, 2015). Two other samples (imprisoned and community based) found that a maltreatment history was related to substance abuse during pregnancy (Chung et al., 2010; Fogel & Belyea, 2001). One small study found no association between maltreatment history and problematic alcohol use during the first trimester of pregnancy (Lang et al., 2006).

#### 3.1.5. Suicidal ideation

Two studies investigated the interrelationship between maltreatment history and suicidal ideation during the transition to parenthood. Pregnant women with maltreatment histories who were referred for psychiatric evaluation were significantly more likely to report suicidal ideation compared to those without a maltreatment history (Farber, Herbert, & Reviere, 1996). Postpartum, women with maltreatment histories were at high risk of suicidal ideation at 4 months postpartum (37% of sample), which remained high for the duration of the study at 15 and 18 months postpartum (~25% of the sample; Muzik et al., 2016).

### 3.2. Physical changes

Fourteen studies in the current review investigated physical changes experienced by prospective and new parents with maltreatment histories.

#### 3.2.1. Pregnancy and birth experience

Women with maltreatment histories were more likely to experience adverse pregnancy and birth experiences (Bernazzani & Bifulco, 2003). A maltreatment history was associated with high rates of unintended first pregnancy (Dietz et al., 1999). Moreover, a maltreatment history was related to premature birth (Christiaens, Hegadoren, & Olson, 2015; Leeners et al., 2014) and low birth weight (Cederbaum, Putnam-Hornstein, King, Gilbert, & Needell, 2013).

#### 3.2.2. Bodily experiences during pregnancy and post-birth

Women with a maltreatment history were found to report greater pain intensity in late pregnancy. However, this association was not significantly different from women without a maltreatment history (Drevin et al., 2015). In addition, a maltreatment history was related to dissociation during pregnancy (Lev-Wiesel, Daphna-Tekoah, & Hallak, 2009) and in labour (Seng et al., 2013; Choi & Seng, 2016). Compared to mothers without maltreatment histories, mothers with maltreatment histories were equally likely to initiate breastfeeding, but more likely to cease exclusive breastfeeding earlier (Ukah, Adu, De Silva, & von Dadelszen, 2016).

#### 3.2.3. Endocrinological differences

A maltreatment history was also associated with hypothalamic–pituitary–adrenal axis (dys)functions. Lower baseline cortisol concentrations were found in pregnant women with maltreatment histories (Shea et al., 2007) and in infants of mothers with a maltreatment history (Brand et al., 2010). Severity of childhood violence exposure in mothers was correlated with reduced pre-stress cortisol levels in a play paradigm involving separations and reunions (Schechter et al., 2004), but not to cortisol reactivity.

Mothers with a maltreatment history showed increased cortisol response and steeper cortisol decline while watching their infant undergoing a stress test (Brand et al., 2010). Pregnant women with sexual abuse histories showed higher evening and morning cortisol levels after stressful days (Bublitz & Stroud, 2013). A larger sample of pregnant women found that current general family functioning significantly moderated the effect of severity of sexual abuse history on increased cortisol awakening response (Bublitz, Stephanie, & Stroud, 2014). Another study identified higher levels of diurnal cortisol as a mediator: childhood maltreatment was related to higher levels of cortisol, which, in turn, were related to less maternal sensitivity (Gonzalez, Jenkins, Steiner, & Fleming, 2012).

### 3.3. Parental view of the child

Fifteen studies provided information on mothers’ perception of their child. Central issues were bonding and attachment, maladaptive parent–child interactions, mother’s perception of (ambiguous) behaviour, and attitudes towards children and parenting.

#### 3.3.1. Bonding and attachment

Mothers with a maltreatment history experienced difficulty bonding with their child (Milan, Lewis, Ethier, Kershaw, & Ickovics, 2004). Specifically, the impairment of reflective functioning in mothers with maltreatment histories was associated with a disorganized attachment style in children (Berthelot et al., 2015). These effects were mainly driven by mental health problems (Roberts et al., 2004) or PTSS (Muzik et al., 2013), or mediated by maternal health status (Seng et al., 2013).

#### 3.3.2. Maladaptive parent–child interactions

Maladaptive interactions are characterized by reduced sensitivity and availability behaviours during exchanges with the child, as well as increased intrusiveness or levels of aversion (Lyons-Ruth & Block, 1986). Emotional abuse in childhood was more predictive of maladaptive mother–infant interactions, compared to physical abuse (Lang et al., 2010). Similarly, mothers with maltreatment histories were less able to establish a healthy emotional connection with their child in the first 12 months postpartum, compared to mothers without maltreatment histories (Fuchs, Möhler, Resch, & Kaess, 2015). Mothers with maltreatment histories displayed increased negative affect during interactions with their child (Lyons-Ruth, Zoll, Connell, & Grunebaum, 1989; Morelen, Menke, Rosenblum, Beeghly, & Muzik, 2016).

#### 3.3.3. Mother’s perception of child behaviour

A physical neglect history was related to inconsistent and inappropriate expectations of the child (e.g. the desire to be taken care of by the infant; Herzog, Gara, & Rosenberg, 1992; Malone, Levendosky, Dayton, & Bogat, 2010). Mothers with maltreatment histories tended to negatively perceive unknown infant faces (Dayton, Huth-Bocks, & Busuito, 2016), were found to be more aggressive towards their own children (Berlin, Appleyard, & Dodge, 2011), and perceived their child’s temperament as more ‘difficult’ (Casanueva et al., 2010).

#### 3.3.4. Attitudes towards children and parenting

Mothers with severe maltreatment histories reported more positive attitudes towards corporal punishment, which was associated with mothers being twice as likely to spank their infant, compared to mothers without maltreatment histories (Chung et al., 2009). A sample of incarcerated pregnant women with maltreatment histories reported attitudes indicative of risk for poor parenting and abuse, such as insensitive behaviour and not valuing the child’s emotional needs (Fogel & Belyea, 2001). However, differences in parenting style between mothers with and without maltreatment histories have not been found consistently (Sexton et al., 2017).

### 3.4. View of the self as a parent

Thirteen studies focused on the impact of maltreatment histories on the perception of self as a parent, and the parenting experience.

#### 3.4.1. Self-perception and identity

Findings from a qualitative study found that mothers with maltreatment histories viewed the transition to parenthood as a new beginning, with specific themes emerging around identity as a mother, hopes and dreams for the future, and love for their children (Aparicio, Pecukonis, & O’Neale, 2015). A case study posited that mothers with maltreatment histories who could integrate perceptions of themselves as abused children into their overall structure as a parent would have more empathy towards their children (Herzog et al., 1992). Parents with maltreatment histories reported re-experiencing repressed feelings of innocence and vulnerability through their child when becoming parents themselves (Erdmans & Black, 2008). While a maltreatment history does not seem to have an association with attitudes towards pregnancy (Altemeier, O’Connor, Sherrod, Tucker, & Vietze, 1996), mothers with severe maltreatment histories (who had experienced more than one type of maltreatment) reported significantly greater levels of self-criticism (Michl et al., 2015) and lower self-esteem (Altemeier et al., 1996; Esparza, 1996).

#### 3.4.2. Subjective view of parenting

Maternal resilience, rather than a maltreatment history, was found to predict self-reported parental competence (Sexton et al., 2015). Some mothers with maltreatment histories qualitatively reported parenting-specific positive post-traumatic change (e.g. ‘most parents would yell, scream … but since I’ve been there, that’s the last thing you want to do’; Fava et al., 2016, p. 24). However, several long-term negative outcomes of a maltreatment history on the transition to parenthood were also identified. In the same qualitative research, mothers with more severe maltreatment histories reported negative parenting-specific post-traumatic change (e.g. ‘I don’t really have my own coping mechanism so it’s really hard to teach him …’; Fava et al., 2016, p. 24). In addition, mothers with maltreatment histories reported their parenting behaviours as being less effective (Caldwell et al., 2011; Michl et al., 2015), and experienced reduced feelings of control, while increasing attributions of child control over problematic events (Bernstein, Laurent, Measelle, Hailey, & Ablow, 2013). Mothers with maltreatment histories also reported increased parenting stress (Shenk et al., 2017), which was mediated by depressive symptoms. One study reported that a maltreatment history without postpartum psychopathology had no negative effect on self-reported maternal parenting behaviour (Muzik et al., 2013).

## 4. Discussion

The aim of this paper was to identify and synthesize the current evidence base on the transition to parenthood following a childhood maltreatment history. The theoretical frameworks by Raphael-Leff (2001) and Stern and Bruschweiler-Stern (1998) were combined, and the overarching theme of mental health was included for this purpose.

The findings from the current review suggest many salient aspects of transitioning to parenthood within the first two years of the child’s life for parents with a maltreatment history. During the transition to parenthood, parents with a maltreatment history appear to be at greater risk of mental health problems and impaired parent–child relationships. Of note, the literature has primarily focused on mothers’ experiences of this transition rather than fathers’, and on parents in high-income countries, as opposed to low- and middle-income countries.

Women with a maltreatment history are more at risk for developing mental health difficulties during their pregnancy and postpartum (Nagl, Lehning, Stepan, Wagner, & Kersting, 2017). This can negatively impact parenting outcomes such as parenting satisfaction, and the parent–child relationship (Berg-Nielsen et al., 2002). Evidence from the review suggests that mental health underpins the experience of physical changes, parental view of the child, and view of self as a parent, across the perinatal and postpartum period. It should be noted that while a maltreatment history is a risk factor for mental health problems during the transition to parenthood, identified mediating factors indicate that there are other important factors to consider: Social support, coping strategies, and resilience. These factors may provide a focus for future interventions. However, caution should be taken with the interpretation of results, as some samples targeted populations where there is a risk of confounding factors that have been previously related to mental health problems (e.g. young age or low socioeconomic status; see Lipman, Offord, & Boyle, 1997). Although difficult to disentangle, considering the high rates of mental health problems consistently found across different studies, childhood maltreatment may be considered a risk factor for mental health problems during the transition to parenthood.

Findings from the current review show that mothers with maltreatment histories (and their children) are at risk of adverse physical changes from conception to birth. This finding is supported in the wider literature, in that decreased cortisol levels have been found in women who have experienced physical/sexual childhood abuse (Meewisse, Reitsma, de Vries, Gersons, & Olff, 2007). Psychological distress may also play a role; for instance, earlier cessation of breastfeeding may be due to breastfeeding triggering distress (Klingelhafer, 2007). It is important for clinicians to be sensitive to women with a maltreatment history given the risk for adverse physical changes across the pregnancy and birthing periods.

Mothers’ views of their child during their transition to parenthood were consistent with the wider literature on distortions of perspectives on children among parents with maltreatment histories (Howe, 2010). These distortions could lead to mother–child role reversal, in which mothers with maltreatment histories become dependent on their child to support their own emotional needs (DiLillo & Damashek, 2003). Furthermore, this role reversal may invite negative parenting behaviours such as aggressiveness and reduced sensitivity. Again, there are confounding risk factors for this finding, including low education levels (Romano, Babchishin, Marquis, & Frechette, 2015) and higher levels of cortisol (Gonzalez et al., 2012).

There were conflicting results regarding mothers’ views of themselves as parents. The experiences of prospective parents during the transition to parenthood, as well as their new identities as parents, are important for positive parenting and bonding with their child (Olde, van der Hart, Kleber, & van Son, 2006). However, a maltreatment history may impair the successful integration of a new parent’s identity. Contextual factors need to be taken into consideration, as it is possible that mothers with maltreatment histories have fewer resources (e.g. social support, modelling of healthy relationships, emotional regulation, and reflective functioning) to draw on. Although these mothers are likely to have fewer resources, they can still express positivity about becoming a parent. Reports of positive parenting change following maltreatment indicate that the transition could be important for personal recovery and growth. Further research could investigate areas such as post-traumatic growth and resilience, as these factors could be related to more positive mental health, physical changes, and perceptions of the child.

A research bias towards mothers was evident in the review. Only one study focused on the impact of childhood maltreatment on fathers during their partners’ pregnancy, which found that fathers with maltreatment histories were also at risk of developing psychopathological symptoms during the tran-sition to parenthood (Skjothaug et al., 2015). Understanding the father’s experience is important because paternal involvement can have important psychological and emotional outcomes in the early years of child development (Opondo, Redshaw, Savage-McGlynn, & Quigley, 2016). Despite the overlap, fathers and mothers experience different pressures and have differing perspectives of their early parenting roles (for a review, see Nystrom & Ohrling, 2004). Future research should investigate these difficulties across mothers and fathers in more depth to ensure that support is tailored to individual needs.

### 4.1. Limitations

Findings from the current review should be considered in the context of its limitations. While the literature search was not constrained, the screening process and subsequent synthesis of evidence were restricted by a theoretical framework. Although the framework provided a theoretical lens through which to understand the current evidence base, and was expanded to include mental health manifestations, findings from our scoping review may not be exhaustive. Research investigating the transition to parenthood following childhood maltreatment was excluded if the results were inconsistent with the theoretical framework categories. In addition, only articles published in English were included. The current review was a qualitative synthesis and the results are limited by the lack of quality assessment of studies and our inability to compare the relative effects of childhood maltreatment owing to variations in study design and methodology. Lastly, it must be noted that our literature search used only four databases, which may not have produced an exhaustive literature search.

### 4.2. Clinical implications

Childhood maltreatment is, unfortunately, a common experience (Stoltenborgh et al., 2015). While the long-term impact of childhood maltreatment varies, expectant parents with a maltreatment history are at increased risk for negative mental health outcomes. We suggest that trauma-informed training should be offered to medical professionals working with prospective parents who may have a history of maltreatment. Prospective and new parents with maltreatment histories may benefit from being able to express their needs to medical professionals and receiving tailored care.

Screening for adverse childhood experiences has been suggested and introduced in other areas owing to the increased risks of negative mental health outcomes and undesirable physical health outcomes associated with childhood maltreatment (Finkelhor, 2017). However, we contend that screening for childhood maltreatment among expectant parents is premature and should be discouraged unless adequate responses or interventions have been developed, tested, and funded, and are ready to be deployed through specified pathways of delivery.

## 5. Conclusion

Considerable research effort has been made to understand the risk factors for maltreatment and consequences on child development. Findings from this review highlight the complicated and complex nature of transitioning to parenthood with a maltreatment history. Overall, a maltreatment history has a negative impact for parents across the prenatal period and up to two years postpartum. Prospectively, research attention could incorporate resilience views of survivors of childhood maltreatment, such as the transition to parenthood as a new beginning for themselves.

## Supplementary Material

Supplemental Material

## Notes

*The full reference list (including those included in the scoping review) has been provided as supplementary material.
