# Supplementary material for: The transition to parenthood following a history of childhood maltreatment: a review of the literature on prospective and new parents’ experiences
Source: Eur J Psychotraumatol. 2018 Aug 2;8(Suppl 7):1492834. doi: 10.1080/20008198.2018.1492834 (PMC7803084; doi:10.1080/20008198.2018.1492834)
Supplement: Supplemental Material [file ZEPT_A_1492834_SM2052.docx]

**Supplementary Material**

**References (of those included in the scoping review)**

Altemeier, W. A., O'Connor, S., Sherrod, K. B., Tucker, B. A., & Vietze, P. (1996). Outcome of Abuse During Childhood Among Pregnant Low Income Women. *Child Abuse and Neglect, 10*(3), 319 - 330. doi: 10.1016/0145-2134(86)90007-4

Aparicio, E., Pecukonis, E. V., & O'Neale, S. (2015). “The love that I was missing”: Exploring the lived experience of motherhood among teen mothers in foster care. *Child Youth Serv Rev, 51*, 44-54. doi: 10.1016/j.childyouth.2015.02.002

Berlin, L. J., Appleyard, K., & Dodge, K. A. (2011). Intergenerational continuity in child maltreatment: Mediating mechanisms and implications for prevention. *Child Dev, 82*(1), 162-176. doi: 10.1111/j.1467-8624.2010.01547.x

Bernazzani, O., & Bifulco, A. (2003). Motherhood as a Vulnerability Factor in Major Depression: The Role of Negative Pregnancy Experiences. *Social Science & Medicine, 56*(6), 1249 - 1260. doi: 10.1016/S0277-9536(02)00123-5

Bernstein, R. E., Laurent, H. K., Measelle, J. R., Hailey, B. C., & Ablow, J. C. (2013). Little Tyrants or Just Plain Tired: Evaluating Attributions for Caregiving Outcomes Across the Transition to Parenthood. *Journal of Family Psychology, 27*(6), 851 - 861. doi: 10.1037/a0034651

Berthelot, N., Ensink, K., Bernazzani, O., Normandin, L., Luyten, P., & Fonagy, P. (2015). Intergenerational transmission of attachment in abused and neglected mothers: The role of trauma‐specific reflective functioning. *Infant Ment Health J, 36*(2), 200-212. doi: 10.1002/imhj.21499

Bouvette-Turcot, A. A., Fleming, A. S., Wazana, A., Sokolowski, M. B., Gaudreau, H., Gonzalez, A., . . . Meaney, M. J. (2015). Maternal childhood adversity and child temperament: an association moderated by child 5-HTTLPR genotype. *Genes Brain Behav, 14*(3), 229-237. doi: 10.1111/gbb.12205

Bouvette-Turcot, A-A., Unternaehrer, E., Gaudreau, H., Lydon, J. E., & Meaney, M. J. (2017). The joint contribution of maternal history of early adversity and adulthood depression to socioeconomic status and potential relevance for offspring development. *Journal of Affective Disorders, 207,* 26-31. doi: [10.1016/j.jad.2016.08.012](https://doi.org/10.1016/j.jad.2016.08.012)

Brand, S. R., Brennan, P. A., Newport, D. J., Smith, A. K., Weiss, T. E., & Stowe, Z. N. (2010). The impact of maternal childhood abuse on maternal and infant HPA axis function in the postpartum period. *Psychoneuroendocrinology, 35*(5), 686-693. doi: 10.1016/j.psyneuen.2009.10.009

Bublitz, M. H., & Stroud, L. R. (2013). Maternal history of child abuse moderates the association between daily stress and diurnal cortisol in pregnancy: A pilot study. *Stress, 16*(6), 706–710. doi:10.3109/10253890.2013.825768

Bublitz, M. H., Parade, S., & Stroud, L. R. (2014). The effects of childhood sexual abuse on cortisol trajectories in pregnancy are moderated by current family functioning. *Biological Psychology, 103,* 152-7. doi: 10.1016/j.biopsycho.2014.08.014

Caldwell, J. G., Shaver, P. R., Li, C.-S., & Minzenberg, M. J. (2011). Childhood maltreatment, adult attachment, and depression as predictors of parental self-efficacy in at-risk mothers. *Journal of Aggression, Maltreatment & Trauma, 20*(6), 595-616. doi: 10.1080/10926771.2011.595763

Casanueva, C., Goldman-Fraser, J., Ringeisen, H., Lederman, C., Katz, L., & Osofsky, J. D. (2010). Maternal perceptions of temperament among infants and toddlers investigated for maltreatment: Implications for services need and referral. *Journal of Family Violence, 25*(6), 557-574. doi: 10.1007/s10896-010-9316-6

Cederbaum, J. A., Putnam-Hornstein, E., King, B., Gilbert, K., & Needell, B. (2013). Infant birth weight and maltreatment of adolescent mothers. *American Journal of Preventive Medicine, 45*, 197-201. doi: 10.1016/j.amepre.2013.03.016

Choi, K. R., & Seng, J. S. (2016). Predisposing and precipitating factors for dissociation during labor in a cohort study of posttraumatic stress disorder and childbearing outcomes. *Journal of Midwifery & Women's Health, 61*(1), 68-76. doi: 10.1111/jmwh.12364

Choi, K. W., Sikkema, K. J., Velloza, J., Marais, A., Jose, C., Stein, D. J., . . . Joska, J. A. (2015). Maladaptive coping mediates the influence of childhood trauma on depression and PTSD among pregnant women in South Africa. *Arch Womens Ment Health, 18*(5), 731-738. doi: 10.1007/s00737-015-0501-8

Christiaens, I., Hegadoren, K., & Olson, D. M. (2015). Adverse childhood experiences are associated with spontaneous preterm birth: a case-control study. *BMC Med, 13*, 124. doi: 10.1186/s12916-015-0353-0

Chung, E. K., Mathew, L., Rothkopf, A. C., Elo, I. T., Coyne, J. C., & Culhane, J. F. (2009). Parenting attitudes and infant spanking: the influence of childhood experiences. *Pediatrics, 124*(2), e278-286. doi: 10.1542/peds.2008-3247

Chung, E. K., Nurmohamed, L., Mathew, L., Elo, I. T., Coyne, J. C., & Culhane, J. F. (2010). Risky health behaviors among mothers-to-be: the impact of adverse childhood experiences. *Acad Pediatr, 10*(4), 245-251. doi: 10.1016/j.acap.2010.04.003

Dayan, J. C., Creveuil, C., Dreyfus, M., Herlicoviez, M., Baleyte, J. M., & O'Keane, V. (2010). Developmental model of depression applied to prenatal depression: role of present and past life events, past emotional disorders and pregnancy stress. *PLoS One, 5*(9), e12942. doi: 10.1371/journal.pone.0012942

Dayton, C. J., Huth-Bocks, A. C., & Busuito, A. (2016). The influence of interpersonal aggression on maternal perceptions of infant emotions: Associations with early parenting quality. *Emotion, 16*(4), 436-448. doi: 10.1037/emo0000114

Dietz, P. M., Spitz, A. M., Anda, R. F., Williamson, D. F., McMahon, P. M., Santelli, J. S., . . . Kendrick, J. S. (1999). Unintended pregnancy among adult women exposed to abuse or household dysfunction during their childhood. *JAMA, 282*(14), 1359-1364.

Drevin, J., Stern, J., Annerback, E. M., Peterson, M., Butler, S., Tyden, T., . . . Kristiansson, P. (2015). Adverse childhood experiences influence development of pain during pregnancy. *Acta Obstet Gynecol Scand, 94*(8), 840-846. doi: 10.1111/aogs.12674

Dym Bartlett, J., & Easternrooks, M. A. (2015). The moderating effect of relationships on intergenerational risk for infant neglect by young mothers. *Child Abuse and Neglect, 45* 21-34. doi: 10.1016/j.chiabu.2015.02.018

Erdmans, M. P., & Black, T. (2008). What they tell you to forget: From child sexual abuse to adolescent motherhood. *Qualitative Health Research, 18*(1), 77-89. doi: 10.1177/1049732307309004

Esparza, D. V., & Esperat, M. C. (1996). The effects of childhood sexual abuse on minority adolescent mothers. *J Obstet Gynecol Neonatal Nurs, 25*(4), 321-328

Farber, E. W., Herbert, S. E., & Reviere, S. L. (1996). Childhood abuse and suicidality in obstetrics patients in a hospital-based urban prenatal clinic. *General Hospital Psychiatry, 18*(1), 56-60. doi: 10.1016/0163-8343(95)00098-4

Fava, N. M., Simon, V. A., Smith, E., Khan, M., Kovacevic, M., Rosenblum, K. L., Menke, R., & Muzik, M. (2016). Perceptions of general and parenting-specific posttraumatic change among postpartum mothers with histories of childhood maltreatment. *Child Abuse Negl, 56*, 20-29. doi: 10.1016/j.chiabu.2016.04.007

Fogel, C. I., & Belyea, M. (2001). Psychological risk factors in pregnant inmates: A challenge for nursing. *MCN: The American Journal of Maternal/Child Nursing, 26*(1), 10-16. doi: 10.1097/00005721-200101000-00004

Frankenberger, D. J., Clements-Nolle, K., & Yang, W. (2015). The Association between Adverse Childhood Experiences and Alcohol Use during Pregnancy in a Representative Sample of Adult Women. *Womens Health Issues, 25*(6), 688-695. doi: 10.1016/j.whi.2015.06.007

Fuchs, A., Möhler, E., Resch, F., & Kaess, M. (2015). Impact of a maternal history of childhood abuse on the development of mother–infant interaction during the first year of life. *Child Abuse Negl, 48*, 179-189. doi: 10.1016/j.chiabu.2015.05.023

Gara, M. A., Allen, L. A., Herzog, E. P., & Woolfolk, R. L. (2000). The abused child as parent: the structure and content of physically abused mothers' perceptions of their babies. *Child Abuse and Neglect, 24*(5), 627-639. doi: 10.1016/S0145-2134(00)00130-7

Gonzalez, A., Jenkins, J. M., Steiner, M., & Fleming, A. S. (2012). Maternal early life experiences and parenting: The mediating role of cortisol and executive function. *Journal of the American Academy of Child & Adolescent Psychiatry, 51*(7), 673-682. doi: 10.1016/j.jaac.2012.04.003

Herzog, E. P., Gara, M. A., & Rosenberg, S. (1992). The abused child as parent: perception of self and other. *Infant Ment Health J, 13*(1), 83-98. doi: 10.1002/1097-0355(199221)13:1<83::AID IMHJ2280130111>3.0.CO;2-6

Huth-Bocks, A. C., Krause, K., Ahlfs-Dunn, S., Gallagher, E., & Scott, S. (2013). Relational Trauma and Posttraumatic Stress Symptoms among Pregnant Women. *Psychodynamic Psychiatry, 41*(2), 277-301. doi: 10.1521/pdps.2013.41.2.277.

Kettunen, P., & Hintikka, J. (2017). Psychosocial risk factors and treatment of new onset and recurrent depression during the post-partum period. *Nord J Psychiatry*, 1-7. doi: 10.1080/08039488.2017.1300324

Lang, A. J., Gartstein, M. A., Rodgers, C. S., & Lebeck, M. M. (2010). The impact of maternal childhood abuse on parenting and infant temperament. *Journal of Child and Adolescent Psychiatric Nursing, 23*(2), 100-110. doi: 10.1111/j.1744-6171.2010.00229.x

Lang, A. J., Rodgers, C. S., & Lebeck, M. M. (2006). Associations between maternal childhood maltreatment and psychopathology and aggression during pregnancy and postpartum. *Child Abuse Negl, 30*(1), 17-25. doi: 10.1016/j.chiabu.2005.07.006

Leeners, B., Rath, W., Block, E., Gorres, G., & Tschudin, S. (2014). Risk factors for unfavorable pregnancy outcome in women with adverse childhood experiences. *J Perinat Med, 42*(2), 171-178. doi: 10.1515/jpm-2013-0003

Lev-Wiesel, R., Daphna-Tekoah, S., & Hallak, M. (2009). Childhood Sexual Abuse as a Predictor of Birth-Related Posttraumatic Stress and Postpartum Posttraumatic Stress. *Child Abuse and Neglect, 33*(12), 877 - 887. doi: 10.1016/j.chiabu.2009.05.004

Li, Y., Long, Z., Cao, D., & Cao, F. (2017). Maternal history of child maltreatment and maternal depression risk in the perinatal period: A longitudinal study. *Child Abuse Negl, 63*, 192-201. doi: 10.1016/j.chiabu.2016.12.001

Lyons-Ruth, K., & Block, D. L. (1996). The disturbed caregiving system: relations among childhood trauma, maternal caregiving, and infant affect and attachment. *Infant Ment Health J, 17*(3), 257-275. doi: 10.1002/(SICI)1097-0355(199623)17:3<257::AID-IMHJ5>3.0.CO;2-L

Lyons-Ruth, K., Zoll, D., Connell, D., & Grunebaum, H. U. (1989). Family deviance and family disruption in childhood: Associations with maternal behavior and infant maltreatment during the first two years of life. *Dev Psychopathol, 1*(3), 219-236. doi: 10.1017/S0954579400000420

Malone, J. C., Levendosky, A. A., Dayton, C. J., & Bogat, G. A. (2010). Understanding the "ghosts in the nursery" of pregnant women experiencing domestic violence: Prenatal maternal representations and histories of childhood maltreatment. *Infant Ment Health J, 31*(4), 432-454. doi: 10.1002/imhj.20264

Marcenko, M. O., Kemp, S. P., & Larson, N. C. (2000). Childhood experiences of abuse, later substance use, and parenting outcomes among low-income mothers. American Journal of Orthopsychiatry, 70(3), 316-326. doi: 10.1037/h0087853.

Martinez-Torteya, C. D., Carolyn J.: Beeghly, Marjorie: Seng, Julia S.: McGinnis, Ellen: Broderick, Amanda: Rosenblum, Katherine: Muzik, Maria. (2014). Maternal parenting predicts infant biobehavioral regulation among women with a history of childhood maltreatment. *Dev Psychopathol, 26*(2), 379-392. doi: 10.1017/S0954579414000017

Marysko, M., Reck, C., Mattheis, V., Finke, P., Resch, F., & Moehler, E. (2010). History of childhood abuse is accompanied by increased dissociation in young mothers five months postnatally. *Psychopathology, 43*(3), 1049. doi: 10.1159/000276999.

McDonnell, C. G., & Valentino, K. (2016). Intergenerational effects of childhood trauma: Evaluating pathways among maternal ACEs, perinatal depressive symptoms, and infant outcomes. *Child Maltreat, 21*(4), 317-326. doi: 10.1177/1077559516659556

Michl, L. C., Handley, E. D., Rogosch, F., Cicchetti, D., & Toth, S. L. (2015). Self-criticism as a mechanism linking childhood maltreatment and maternal efficacy beliefs in low-income mothers with and without depression. *Child Maltreat, 20*(4), 291-300. doi: 10.1177/1077559515602095

Milan, S., Lewis, J., Ethier, K., Kershaw, T., & Ickovics, J. R. (2004). The Impact of Physical Maltreatment History on the Adolescent Mother-Infant Relationship: Mediating and Moderating Effects During the Transition to Early Parenthood. *J Abnorm Child Psychol, 32*(3), 249-261. doi: 10.1023/B:JACP.0000026139.01671.fd

Morelen, D., Menke, R., Rosenblum, K. L., Beeghly, M., & Muzik, M. (2016). Understanding bidirectional mother-infant affective displays across contexts: Effects of maternal maltreatment history and postpartum depression and PTSD symptoms. *Psychopathology, 49*(4), 305-314. doi: 10.1159/000448376

Muzik, M., Bocknek, E. L., Broderick, A., Richardson, P., Rosenblum, K. L., Thelen, K., & Seng, J. S. (2013). Mother-infant Bonding Impairment Across the First 6 Months Postpartum: The Primacy of Psychopathology in Women with Childhood Abuse and Neglect Histories. *Archives of Womens Mental Health, 16*(1), 29 - 38. doi: 10.1007/s00737-012-0312-0

Muzik, M., Brier, Z., Menke, R. A., Davis, M. T., & Sexton, M. B. (2016). Longitudinal suicidal ideation across 18-months postpartum in mothers with childhood maltreatment histories. *Journal of Affective Disorder, 204, 138*-*45. doi:* 10.1016/j.jad.2016.06.037.

Muzik, M., Morelen, D., Hruschak, J., Rosenblum, K. L., Bocknek, E., & Beeghly, M. (2017). Psychopathology and parenting: An examination of perceived and observed parenting in mothers with depression and PTSD. *Journal of Affective Disorders, 207*, 242-250. doi: [10.1016/j.jad.2016.08.035](https://doi.org/10.1016/j.jad.2016.08.035).

Oh, W., Muzik, M., McGinnis, E. W., Hamilton, L., Menke, R. A., & Rosenblum, K. L. (2016). Comorbid trajectories of postpartum depression and PTSD among mothers with childhood trauma history: Course, predictors, processes and child adjustment. *J Affect Disord, 200*, 133-141. doi: 10.1016/j.jad.2016.04.037

Plant, D. T., Barker, E. D., Waters, C. S., Pawlby, S., & Pariante, C. M. (2013). Intergenerational transmission of maltreatment and psychopathology: The role of antenatal depression. *Psychol Med, 43*(3), 519-528.

Roberts, R., O'Connor, T., Dunn, J., & Golding, J. (2004). The effects of child sexual abuse in later family life; mental health, parenting and adjustment of offspring. *Child Abuse Negl, 28*(5), 525-545. doi: 10.1016/j.chiabu.2003.07.006

Schechter, D. S., Zeanah, C. H., Myers, M. M., Brunelli, S. A., Liebowitz, M. R., Marshall, R. D., . . . Hofer, M. A. (2004). Psychobiological dysregulation in violence-exposed mothers: salivary cortisol of mothers with very young children pre- and post-separation stress. *Bulletin of the Menninger Clinic, 68*(4), 319-336. doi: 10.1521/bumc.68.4.319.56642

Seng, J. S., Sperlich, M., Low, L. K., Ronis, D. L., Muzik, M., & Liberzon, I. (2013). Childhood abuse history, posttraumatic stress disorder, postpartum mental health, and bonding: A prospective cohort study. *Journal of Midwifery & Women's Health, 58*(1), 57-68. doi: 10.1111/j.1542-2011.2012.00237.x

Seng, J. S., Sperlich, M., Low, L. K., Ronis, D. L., Muzik, M., & Liberzon, I. (2013). Childhood abuse history, posttraumatic stress disorder, postpartum mental health, and bonding: a prospective cohort study. *Journal of Midwifery and Women’s Health, 58*(1), 57-68. doi: 10.1111/j.1542-2011.2012.00237.x

Sexton, M. B., Hamilton, L., McGinnis, E. W., Rosenblum, K. L., & Muzik, M. (2015). The roles of resilience and childhood trauma history: Main and moderating effects on postpartum maternal mental health and functioning. *J Affect Disord, 174*, 562-568. doi: 10.1016/j.jad.2014.12.036

Sexton, M. B., Davis, M. T., Menke, R., Raggio, G. A., & Muzik, M. (2017). Mother-child interactions at six months postpartum are not predicted by maternal histories of abuse and neglect or maltreatment type. *Psychological Trauma, 9*(5), 622-626. doi: 10.1037/tra0000272

Shea, A. K., Streiner, D. L., Fleming, A., Kamath, M. V., Broad, K., & Steiner, M. (2007). The effect of depression, anxiety and early life trauma on the cortisol awakening response during pregnancy: preliminary results. *Psychoneuroendocrinology*, *32*(8-10), 1013-20.

Shenk, C. E., Ammerman, R. T., Teeters, A. R., Bensman, H. E., Allen, E. K., Putnam, F. W., & Ginkel, J. B. (2017). History of maltreatment in childhood and subsequent parenting stress in at-risk, first-time mothers: Identifying points of intervention during home visiting. *Prevention Science*, No Pagination Specified. doi: 10.1007/s11121-017-0758-4

Skjothaug, T., Smith, L., Wentzel-Larsen, T., & Moe, V. (2015). Prospective fathers' adverse childhood experiences, pregnancy-related anxiety, and depression during pregnancy. *Infant Ment Health J, 36*(1), 104-113. doi: 10.1002/imhj.21485

Stacks, A. M., Muzik, M., Wong, K., Beeghly, M., Huth-Bocks, A., Irwin, J. L., & Rosenblum, K. L. (2014). Maternal reflective functioning among mothers with childhood maltreatment histories: Links to sensitive parenting and infant attachment security. *Attachment & Human Development, 16*(5), 515-533. doi: 10.1080/14616734.2014.935452

Teeters, A. R., Ammerman, R. T., Shenk, C. E., Goyal, N. K., Folger, A. T., Putnam, F. W., & van Ginkel, J. B. (2016). Predictors of maternal depressive symptom trajectories over the first 18 months in home visiting. *American Journal of Orthopsychiatry, 86*(4), 415-424. doi: 10.1037/ort0000159

Ukah, U. V., Adu, P. A., De Silva, D. A., & von Dadelszen, P. (2016). The Impact of a History of Adverse Childhood Experiences on Breastfeeding Initiation and Exclusivity: Findings from a National Population Health Survey. *Breastfeed Med, 11*, 544-550. doi: 10.1089/bfm.2016.0053

van der Waerden, J., Galera, C., Saurel-Cubizolles, M. J., Sutter-Dallay, A. L., & Melchior, M. (2015). Predictors of persistent maternal depression trajectories in early childhood: results from the EDEN mother-child cohort study in France. *Psychol Med, 45*(9), 1999-2012. doi: 10.1017/s003329171500015x
